# Infrared thermography as a possible technique for the estimation of parturition onset in sows

**DOI:** 10.1186/s40813-022-00301-x

**Published:** 2023-02-01

**Authors:** S. M. Gulliksen, T. Framstad, C. Kielland, M. A. Velazquez, M. M. Terøy, E. M. Helland, R. H. Lyngstad, A. J. Oropeza Delgado, M. Oropeza-Moe

**Affiliations:** 1 Norwegian Pig Health Service, Animalia AS, P.O. Box 396, Økern, Norway; 2 Faculty of Veterinary Science, Department of Production Animal Clinical Sciences, Norwegian University of Life Sciences (NMBU), Ås, Norway; 3 School of Natural and Environmental Sciences, Newcastle University, Newcastle Upon Tyne, NE1 7RU UK; 4 Fiskå Mølle AS, Fiskåvegen 1010, 4120 Tau, Norway

**Keywords:** Infrared, Thermography, Rectal, Auricular, Farrowing

## Abstract

**Background:**

This study explores the possibility of using infrared thermography to estimate the onset of parturition in sows. Infrared camera (IRC) and infrared laser thermometer (IRT) were used to obtain the auricular skin temperature of sows along with rectal temperatures, from approximately one week before the anticipated farrowing until 24 h post-partum. Three commercial piglet producing farms were included in the study.

**Results:**

There were large variations in observed auricular skin temperature, both by IRC and IRT per time point. Graphical exploration of the observed auricular skin temperature measured by the two methods showed the same parallel patterns, although temperatures measured by IRC were higher at any time point compared to IRT. Auricular skin thermography revealed a clear increase in temperatures before farrowing. Statistical analyses, adjusting for differences between farms, sow activity and respiration rate, confirmed this increase. When controlling for these variables, and comparing the baseline temperatures to temperatures at farrowing, the difference was 3.9 and 4.1 °C measured with IRT and IRC, respectively. The greatest increase, of more than 2 °C, was found between 16 and 8 h and 8 to 4 h before farrowing. Rectal temperature increased by 0.5 °C in the same time interval and reached a temperature peak after farrowing.

**Conclusion:**

Sows showed a more than 2 °C increase in auricular skin temperature, measured by either IRC or IRT, 8 to16 hours before the first piglet was born. Hence, monitoring auricular skin temperatures of sows using infrared thermography one week before expected farrowing may provide a baseline temperature for each sow from which a sudden rise is indicative of parturition in the following 8 to 16 h. This may lead to more efficient allocation of human assistance during farrowing time and thereby improve farrowing management and the welfare of sows and their offspring.

## Background

Information on individual sows is important for management of their health and welfare. Keeping track of individual sows becomes more challenging as herd sizes increase. Hence, large production units require a high-level of coordination of every aspect of management.

In modern large pig herds, the lack of surveillance at farrowing is a current animal health and welfare challenge. Parturition needs to be closely supervised to minimise losses due to problems with sows or piglets (Edwards and Baxter 2015). Farrowing supervision allows for manual assistance for sows exhibiting dystocia, treatment to help overcome cases of primary and secondary uterine inertia and may also reduce the number of stillbirths (Le Cozler et al. 2002). Farrowing supervision is also vital to ensure sufficient colostrum uptake in the piglets (Quesnel et al. 2012). Today farmers rely on mammary development and behavioural change prior to farrowing as indicators of the farrowing initiation timepoint. One of the clearest signals of impending farrowing is the sow’s increased activity caused by nest building behaviour. For loose housed and outdoor sows, nest building occurs within 24 h before the onset of parturition and is at its most intense 12 to 6 h before farrowing (Algers and Uvnäs-Moberg 2007). Even though crate-confined sows cannot build a nest, they too seem to be highly active the day prior to parturition, performing activities such as pawing, rooting or mouthing the crate fixtures (Rushen et al. 2001; Algers and Uvnäs-Moberg 2007). Detection of nest building activity requires close monitoring of the sows, and as the gestation length in the sow can range from 105 to 125 days, detecting the onset parturition is difficult and time consuming in practice (Sasaki and Koketsu 2007).

To facilitate farrowing supervision, the induction of farrowing by administering the natural hormone prostaglandin F2 alpha (PGF 2 alpha), or a synthetic analogue such as cloprostenol prior to the expected date of farrowing, is recommended in some countries (Peltoniemi and Oliviero 2015). However, human intervention by means of exogenous hormones during parturition includes risks, such as decreased piglet viability, increased risk of dystocia, and reduced sow welfare (Kirkden et al. 2013; Peltoniemi and Oliviero 2015).

Innovative solutions are needed to help monitoring sow behaviour and activity, especially around farrowing. Erez and Hartsock (1990) studied changes in the sow’s body postures prior to parturition using an infrared photocell system mounted on farrowing crates. They reported an increase in frequency of posture changes beginning 12–24 h prior to the birth of the first piglet. By using two kinds of movement sensors to detect the onset of farrowing, Oliviero et al. (2008), concluded that the mean time sows spent standing was significantly higher in the 24-hour interval prior to farrowing than in all the other 24-hour intervals monitored. Cornou et al. (2011) and Pastell et al. (2013) used accelerometers to measure the activity of sows before farrowing and developed models (Cornou and Lundby-Christensen, 2012) to detect the onset of farrowing based on activity changes. The collection of relevant data and the analysis of these data can not only improve animal welfare but also aid in the efficient allocation of human resources and help the stockperson’s focus on those animals which need attention the most.

These methods seem to be reliable but, if combined with other variables that could indicate forthcoming parturition, could be further improved. Accordingly, several studies of rectal thermography and telemetry data indicate that the sow’s body temperature increases just before farrowing, suggesting a possible application for estimation of time of parturition (Bressers et al. 1994; Damgaard et al. 2009; Williams et al. 2013). A passive forced hyperthermia, rather than a rising phase during development of fever, is probably what characterizes the rise in temperature before farrowing (Oka et al. 2001). Hendrix et al. (1978) found an increase in rectal temperature of only 0.4 °C from 12 to 4 h before parturition. Similarly, Kelley and Curtis (1978) reported that rectal temperature increased by 0.6 °C during the 4-hour period prior to parturition in l0 peripartal sows and gilts held at an air temperature of 20.5 °C. In a study of body temperature in sows monitored with an implanted radiotelemetry devices, from 4 to 2 days prepartum to approximately 12 days postpartum, an increase in body temperature of 1.4 °C was associated with parturition (Littledike et al. 1979). For crate-confined sows, Bressers et al. (1994) used a radiotelemetric device implanted under the skin close to the ear base and reported a rise in sows’ temperature starting between 6 and 12 h before farrowing.

Infrared thermography is a non-contact and non-invasive technique used to detect radiation from an object, and, can be used to measure body surface temperature (Ring and Ammer 2012). It does not require restraint or contact with the animal; hence it has the potential of reducing stress levels and disease spread. Infrared thermography is based on electromagnetic radiation, which is characterized by its wavelength and intensity (Schaefer et al. 2004). The wavelength of an object depends on its surface temperature. Infrared temperature measurement equipment is gaining popularity as a diagnostic tool for evaluating human and animal health. It is already applied in veterinary medicine as an ancillary tool for the rapid, automated detection of early disease in animals (Stelletta et al. 2012; Schaefer and Cook 2013; Zhang et al. 2019). It has also been used to detect lameness in horses (Eddy et al. 2001), mastitis in dairy cows (Colak et al. 2008) and calf diarrhoea (Schaefer et al. 2004) as well as for the assessment of animal welfare (Stewart et al. 2005). However, the results from research studying the usefulness of infrared thermography for female reproductive events are less clear. Soede et al. (1997) concluded that changes in vaginal temperature cannot be used as a predictor for ovulation time. On the other hand, several studies have investigated the relationship between vulvar skin temperature and time of ovulation in swine, concluding that the technique could be an aid in predicting time of ovulation (Luño et al. 2013; Scolari, 2010). Likewise, Weng (2020) concluded that infrared thermography could be used for mass screening aimed at the early diagnosis of heat, characterized by an increase of vulvar skin temperature. Still, due to many conflicting parameters as to whether body and/or vaginal temperature is related to the time of oestrus and if those parameters are accurate and reliable, the researchers also state that further studies are needed.

The aim of this study was to determine auricular skin and rectal temperature of sows in the period before, at, and after farrowing, using infrared camera (IRC) and infrared laser thermometer (IRT) in addition to rectal thermography (RT); and investigate the possibility of using infrared thermography to estimate the onset of parturition in sows, and thereby improving farrowing supervision.

## Results

**Fig. 1:**
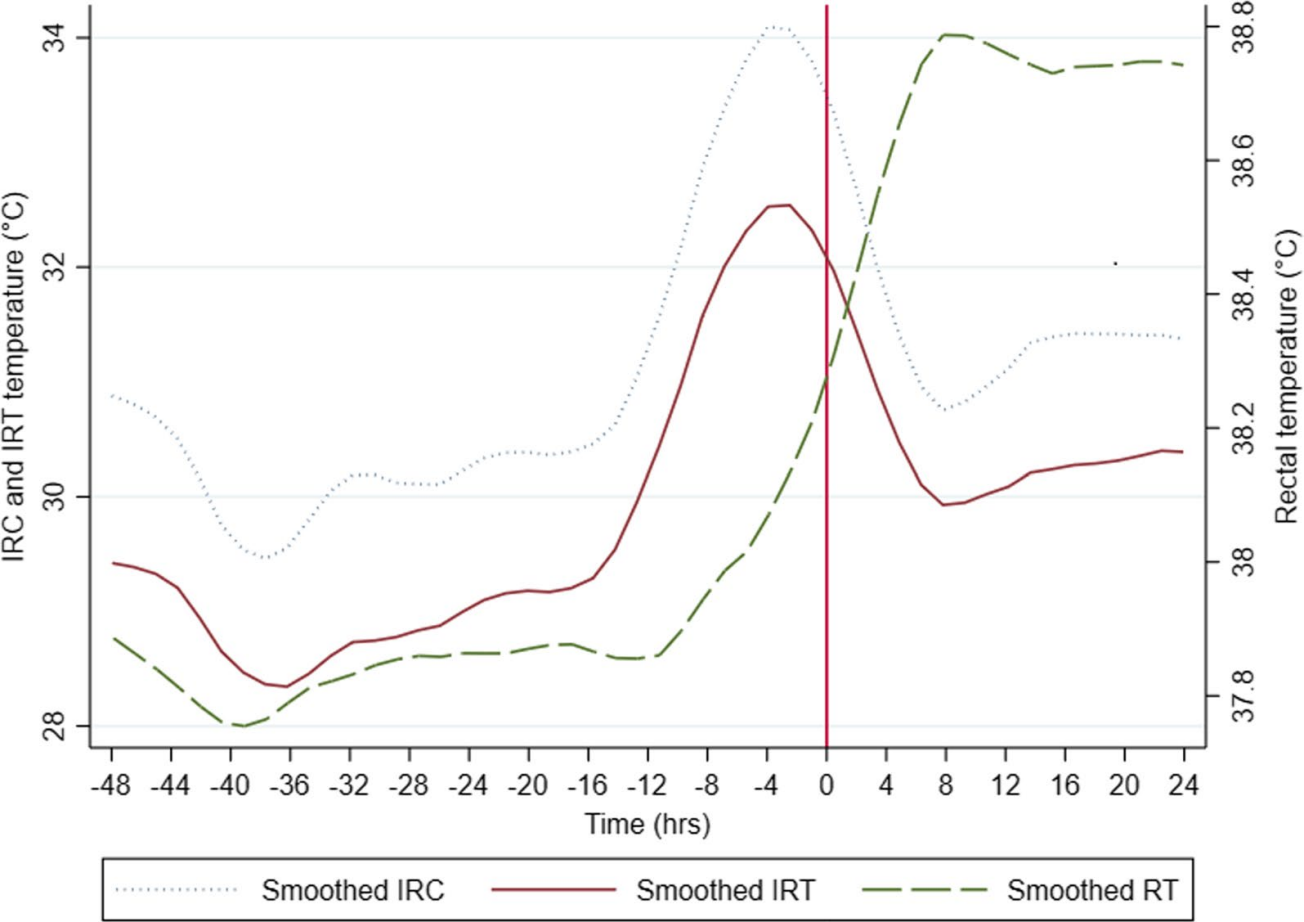
Temperatures before and after farrowing measured by using rectal thermometer, infrared camera and laser thermometer. Legend: Descriptive presentation of body temperature 48 h before to 24 h after farrowing measured by using rectal thermometer (RT, right axis), infrared camera (IRC, left axis) and infrared laser thermometer (IRT, left axis) in 91 sows in three Norwegian piglet producing farms. Time point zero (red vertical line) denotes the birth of the first piglet. The “lpoly” function used performs a kernel-weighted local polynomial univariate regression of the three temperature measurements on the y-axis and time at the x-axis

The observed auricular skin temperature measured from 48 h before to 24 h after farrowing by the two infrared thermography methods showed the same parallel pattern, although temperatures measured by IRC were higher, at any timepoint compared, to IRT (Fig. 1). Rectal temperatures were higher at all timepoints compared to the auricular skin temperatures. Both rectal and auricular skin temperatures measured from 48 h before to 24 h after farrowing showed only small fluctuations until 16 to 8 h before farrowing, when an increase was observed, independent of measuring method used (Table 1). The rise in auricular skin temperature seemed to occur prior to the increase in rectal temperature. Body temperatures measured by rectal thermography reached their highest values shortly after parturition and then remained higher than before parturition, while auricular skin temperature reached a peak before parturition (Fig. 1).

**Table 1:** Observed temperatures before and after farrowing measured by using infrared camera, laser thermometer and rectal thermometer

Hours prior to and post farrowing	IRC		IRT		RT	
	Mean	SD	Mean	SD	Mean	SD
− 48 to > 24	30.2	4.7	28.7	4.4	37.8	0.4
− 24 to > 16	30.5	4.9	29.3	4.5	37.9	0.4
− 16 to > 8	31.0	4.7	29.9	4.4	37.9	0.3
− 8 to > 4	33.7	3.0	32.1	3.5	38.0	0.4
− 4 to > 1	34.3	2.4	32.7	3.0	38.1	0.4
Farrowing	34.3	2.6	32.8	3.0	38.3	0.4
1 to < 4	32.5	3.9	31.4	4.1	38.4	0.5
4 to < 8	31.0	5.2	30.1	4.7	38.8	0.5
8 to < 16	31.0	4.6	30.0	4.3	38.8	0.5
16 to < 24	31.3	4.3	30.2	4.1	38.7	0.5
> 24	31.6	5.0	30.4	4.7	38.6	0.5
Total	31.0	4.7	29.7	4.4	38.0	0.6

Legend: Observed temperature (°C, mean (± SD)) measured by using infrared camera (IRC), infrared laser thermometer (IRT) and rectal thermometer (RT) from 48 h before to 24 h after farrowing in 91 sows in three Norwegian piglet producing farms

There were large variations in measured values of the auricular skin temperature, both by IRC and IRT per time point (Table 1). In the time period from 16 to 8 h prior and up to farrowing, the mean auricular skin temperature was observed to increase by 3.3 °C and 2.9 °C measured by IRC and IRT, respectively. Following farrowing, the sows’ auricular skin temperature dropped. The mean rectal temperature was 0.4 °C higher at farrowing compared to 16 to 8 h before farrowing (Table 1).

Temperature measurements done by IRC and IRT were highly correlated (r = 0.87), while the correlation between rectal temperature and skin temperature measured by IRC and IRT were low (0.08 and 0.09), respectively). Room temperature was highly correlated with both IRC and IRT (r = 0.38 and 0.39), respectively). In comparison, the correlation between room temperature and RT was 0.19. Room temperature was found to explain approximately 15% of the variation in IRC and IRT (R²=0.148 and 0.153 (*P* < 0.001), respectively), while the corresponding influence on RT was 3.0% (*P* < 0.001). Room humidity was negatively correlated with IRC and IRT (r = − 0.17 and − 0.11, (*P* < 0.001), respectively), while the correlation between humidity and RT was 0.16 (*P* < 0.001).

**Fig. 2:**
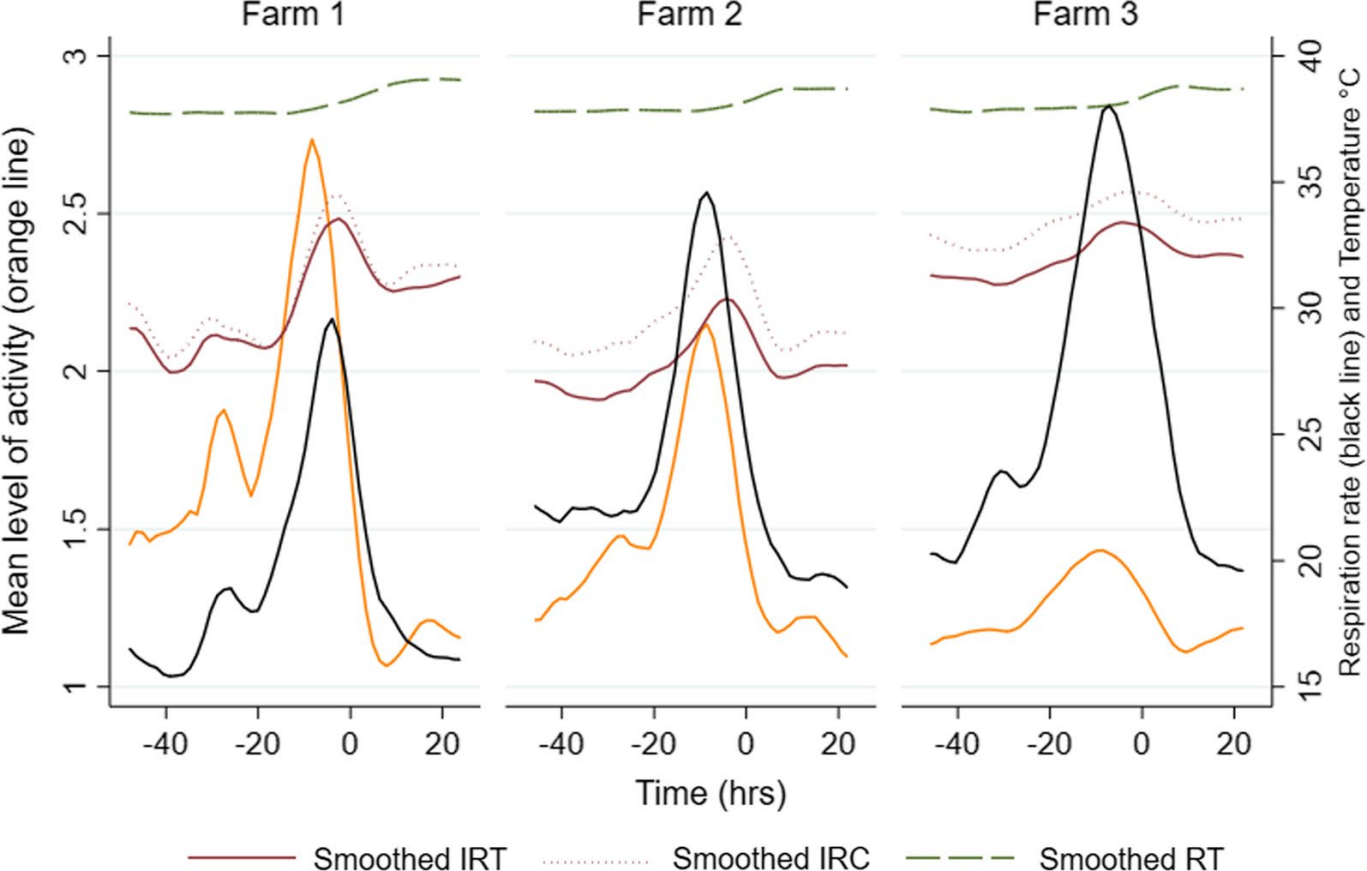
Observed mean activity level, respiration rate and body temperatures before and after farrowing. Legend: Observed mean activity level (left axis, orange line), respiratory rate (right axis, black line), and body temperature (left axis) measured by using rectal thermometer (RT, green line), infrared camera (IRC, red, dotted line) and infrared laser thermometer (IRT, red line) from 48 h before to 24 h after farrowing in 91 either loose housed (Farm 1 and 2) or crated (Farm 3) sows in three Norwegian piglet producing farms. Time point zero denotes the birth of the first piglet. Activity level was recorded as 1: lying down, 2: sitting, 3: standing, or 4: rooting or nest building behaviour. Respiratory rate was recorded as the number of flank movements per minute

Respiratory rate was found to increase from 20 h before farrowing until farrowing, as were the activity levels in the sows at all three farms (Fig. 2). The activity level was lower in crated sows in Farm 3 compared to loose housed sows at the two other farms. Auricular skin temperature seemed to increase as the activity level and respiratory rate increased. The increase in auricular skin temperature was smaller in farm 3 compared to the two other farms. The variation in rectal temperature from 48 h before to 24 h after farrowing was small in all three farms.

**Fig. 3:**
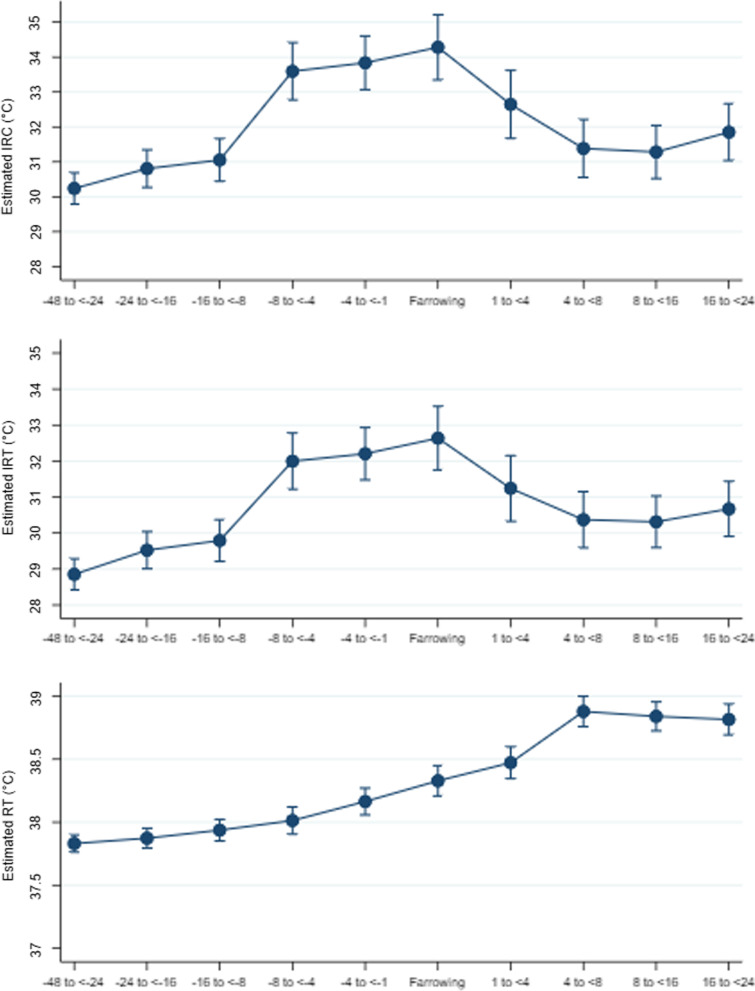
Statistically estimated predictive margins of different temperatures measurements per time period (hours) around farrowing. Legend: Estimated auricular skin temperature measured by Infrared camera thermography (IRC) and Infrared laser thermography (IRT), and rectal temperature (RT) in 91 sows from > 48 h before farrowing until 24 h after farrowing (bars: 95% confidence interval) in three commercial piglet producing farms. The temperature originates from the estimated margins from the mixed model that accounts for repeated measurements on sow level, and for differences between farms, sow activity and the respiration rate at the time of the temperature measurement

As part of the aim of this study was to investigate the possibility of using infrared thermography to estimate the onset of parturition in sows in general, statistical models were used to identify estimated coefficients per timepoint by adjusting for differences between farms (accounting for humidity and room temperature), sow activity and respiration rate. The marginal plots from the best fitted models, and with stratification of farm, confirmed the patterns seen in the observed data, with an increase in temperature before farrowing, and a temperature peak at farrowing for the auricular skin temperature with both infrared methods (Fig. 3). From an estimated baseline auricular skin temperature at 24 to 48 h before farrowing, a temperature-increase until farrowing of 4.1 °C and 3.9 °C was found for IRC and IRT, respectively. The steepest gradient on the curve was found from 16 to 8 h before farrowing until 8 to 4 h before farrowing, where a temperature-increase of 2.5 °C and 2.1 °C measured by IRC and IRT, respectively, was found. The estimated difference in rectal temperature from the baseline temperature until farrowing was 0.5 °C. Rectal temperatures reached their highest values 4 to 8 h after parturition and then remained higher than before parturition. The increase in rectal temperature from 48 h before until 8 h after farrowing was gradual and small compared to the increase in auricular skin temperature.

## Discussion

All sows showed rises in temperature before farrowing, regardless of the measurement method used. Naturally, there were individual variations in body temperature, when the temperature rise started and the extent of the rise. The results of the present work coincide with previous studies in which rectal temperatures of sows were higher than body surface temperatures measured using infrared technology (Malmkvist et al. 2012; Schmidt et al. 2013). A sudden rise in auricular skin temperature measured by infrared thermography (IRC and IRT) of more than 2 °C from 16 to 8 h before farrowing until 8 to 4 h before farrowing has, to our knowledge, not been reported previously. Rectal temperatures reached their highest values during and shortly after parturition and then remained higher than before parturition. This corresponds well with results reported in the literature for temperatures around farrowing measured by rectal thermography (Hendrix et al. 1978; Elmore et al. 1979; Kelley and Curtis 1978) and telemetry (Bressers et al. 1994). Body temperature control in the pig, as in other mammals, is integrated in the hypothalamus (Baldwin and Ingram 1967; Morrison et al. 2008). It has been suggested that the ear surface could react rapidly to temperature changes since the vascular supply to the hypothalamus is near the auricular area (Gomart et al. 2014). This may help explain why the rise in auricular skin temperature, seen in this study, seemed to occur prior to the increase in rectal temperature. While the rise in rectal temperature may provide an additional indication that onset of parturition is near, it is important to note that this increase was gradual and small. Hence, the usability of rectal thermography to estimate the onset of parturition is limited.

The data from the three farms in this study were collected by different people in different years. This may have had an impact on the results. Also, the fact that the auricular skin temperature of each sow was not monitored continuously may have affected the obtained curves. Studies using infrared thermography to estimate body temperature of pigs present somewhat discrepant results, which could be caused by inadequate equipment, varying knowledge about equipment operation, and farm-specific factors not evaluated. In order to reduce the risk of information bias and calibrate the measurements to the greatest extent possible, the veterinary students sampling farm 1 in this study were trained by a veterinarian also involved in the measurements in the other two farms. In addition, all sampling was done during winter in all three farms to reduce the effect of season in the trial.

Skin temperature is the result of complex interactions between environmental factors and the internal body temperature (Malmkvist et al. 2012). Therefore, there might be limitations in the applicability of infrared technology due to factors which could cause a considerable variation in skin temperature, such as variation in behaviour, level of physical activity, variations in the environmental temperature, and the animal’s body condition (Weng 2020). Increasing ambient temperature rapidly increases the skin temperature, due to the vasocontrolled thermoregulation, supplying more blood to the skin tissue (Loughmiller et al. 2001; Malmkvist et al. 2012). Nevertheless, even when including farm as fixed effect, and indirectly the room temperature and humidity, into the fitted model, an increase in skin temperature was seen prior to farrowing. Studies have concluded that the back of the ear is one of the most promising locations for the practical application of infrared methods to measure body temperature (reviewed by Sørensen and Pedersen, 2005; Schmidt et al. 2013), i.e. this area is less affected by the ambient temperature compared to other surface areas of the body. Still, as temperatures outside the thermoneutral area for sows have been shown to influence auricular skin temperature (Loughmiller et al. 2001), variations in the ambient temperature need to be controlled if infrared thermography methods are to be used in practice to predict the onset of parturition. Further studies would have to be conducted in order to assess whether this technology is suitable for farrowing detection in different climate zones. The estimated increase in rectal temperature from 16 to 8 h before farrowing and up until farrowing, accounting for humidity and room temperature, sow activity and respiration rate, was similar to the observed values (0.5 and 0.4 °C, respectively), indicating that the influence of these factors on rectal thermography is minimal. This corresponds to Malmkvist et al. (2012) who found no effect of room temperature on rectal temperature.

The elevated activity level observed from approximately 24 h before farrowing might have influenced both the auricular skin temperature and the rectal temperature in the three farms, as described by Kelley and Curtis (1978). The present study found that the activity level was lower in crated sows in Farm 3 compared to loose housed sows in the two other farms. Even so, the fact that both loose housed and fixed sows showed a significant rise in auricular skin surface temperature before farrowing, when adjusting for farm specifics such as room temperature, indicates that fixation per se and activity limitations for these sows did not prevent an apparent physiological rise in skin temperature.

Wide applications of passive infrared thermography include inflammation detection (Sathiyabarathi et al. 2016; Menzel et al.,2014), ovulation monitoring (Simões et al. 2014; Luño et al. 2013), abnormal behaviour identification (Cook et al. 2018), and growth evaluation (Caldara et al. 2014). Only a few studies have investigated the use of infrared thermography in pigs and even fewer have investigated the use of this method as a tool to identify increases in body temperature (Schmidt et al. 2013; Barbieri et al. 2021). The use of sensor technology can provide more objective and reliable data as the quantity and accuracy of information can contribute to more objective measurements that can assist decision making, as compared to visual assessment (Cornou and Lundbye-Christensen 2012). Also, infrared thermography can detect surface temperature in a non-invasive manner without incurring negative responses from the animal (Stewart et al. 2005; Sørensen and Pedersen 2015). In addition, as it is a non-contact procedure, data can be collected on animals that are difficult to reach or to approach. Furthermore, the short measuring time allows the recording of data from moving animals. Infrared thermography measurements also reduce the risk of spreading infections, since it does not require the subject to be touched. According to current knowledge, single-time point measurements cannot deliver reproducible results under field conditions but implementing multiple measurements for automatical computer software analysis would improve its use by practitioners as a monitoring tool (Schmidt et al. 2013; Peltoniemi and Oliviero 2015).

## Conclusion

In conclusion, sows show a sudden 2 °C increase in auricular skin temperature, 8 to16 hours before the first piglet is born, measured by either an infrared camera or an infrared laser thermometer. Hence, monitoring auricular skin temperatures of sows using infrared thermography one week before expected farrowing may provide a baseline temperature for each sow from which a rise of at least 2 °C will be indicative of parturition in the following 8 to 16 h. This may lead to more efficient allocation of human assistance during farrowing time and thereby improve farrowing management and welfare of sows and their offspring. Further studies are needed to investigate the effects of environmental factors and sow specific factors, such as body condition and parity on skin temperature measured by infrared thermography.

## Methods

### Animals and management

From three piglet producing farms (Farms 1, 2 and 3, respectively), 37 Norwegian Landrace x Yorkshire sows (LY-sows), 27 and 27 Norwegian Landrace x Z-line Yorkshire sows (Topigs Norsvin TN 70 sows) were included in this study (Table 2). An evaluation of overall behaviour and a clinical examination was performed on all sows in order to exclude animals with clinical signs of diseases. Sows medically treated were excluded. A total of 22 (24.2%) of the 91 sows included were first parity sows, while the corresponding numbers for second, third, fourth or more than four parities were 30 (32.9%), 21 (23.1%), 12 (13.2%) and 6 (6.6%), respectively. In all three farms, the sows were moved to the farrowing unit two to three weeks before their expected farrowing date and allocated to individual farrowing pens. Sows were loose housed during the entire production cycle, except from in Farm 3 where the sows were crated in 5.5 days around farrowing on average. The farrowing pens in Farm 1 had solid and slatted floor of 4.5 and 2.8 m², respectively, including a piglet creep area of 0.8 m^2^. The corresponding measures for farrowing pens in Farm 2 and 3 were 5.3 and 1.9 m², and a piglet creep area of 1.1 m². The farrowing pens in Farm 3 were equipped with movable bars, which enabled pens to be modified into crates and vice versa. The sow area (the part of the pen accessible to the sow) for crated sows measured 1.68 m^2^ (0.8 m^2^ × 2.1 m^2^). When the sows were let loose, the sides of the crate were opened and placed along the sidewalls. In all pens in all three farms, there were protection rails along the sidewalls to prevent piglets from being crushed when the sow lay down. As described by Staarvik et al. (2019), all farrowing pens had a creep area with heated floors, in addition to a heat lamp. Water nipples were distributed in two heights, one for the sow and one for piglets. All sows were fed a standard concentrate feed for sows in gestation. Additionally, sows were fed hay daily from the day they were moved to the farrowing unit until farrowing. According to Norwegian legislation, the sows were offered additional straw for nest building at least three days before expected farrowing. In general, the use of farrowing crates does not comply with the Norwegian regulations on swine husbandry (FOR-2003-02-18-175). This trial was accepted by the Norwegian Food Safety Authority (FOTS ID 11,122) due to the potential utility value for increasing welfare in piglet producing farms.

**Table 2:** Characteristics for three Norwegian piglet producing farms included in the study

	Farm 1	Farm 2	Farm 3
Location	Southeastern Norway	Rogaland	Rogaland
Housing	Loose housed	Loose housed	Loose housed but crated during farrowing
Size of farrowing pen	7.3 m²	7.2 m²	7.2 m²
Part of pen accessible for sows during farrowing	6.5 m²	6.1 m²	1.68 m²
Ventilation system	Diffuse	Diffuse	Diffuse
Room temperature °C, mean ± SE	19.4 ± 0.02	18.8 ± 0.04	20.1 ± 0.02
Room humidity %, mean ± SE	66.7 ± 0.17	61.6 ± 0.15	50.6 ± 0.12
Feeding system	Dry	Wet	Wet
Breed	Landrace x Yorkshire	Landrace x Z-line Yorkshire	Landrace x Z-line Yorkshire
Number of sows included	37	27	27
Measurements performed by	Veterinary students	Researchers	Researchers
Year of visit	2013	2016	2017
Time of visit	24.1.–27.1.	16.–22.2.	22.2–3.3.

Legend: Characteristics for three Norwegian piglet producing farms included in the study

### Data collection

Farms 1, 2 and 3 were visited during winter 2013, 2016 and 2017, respectively. Registrations in Farm 1 were mainly performed by veterinary students, while registrations were done by veterinarians in the other two farms. Data were collected approximately one week before the anticipated farrowing date until 24 h after farrowing. For all sows, ID number and parity were registered pre farrowing.

The initiation of farrowing was registered as the time point when the first piglet was born. During the study period, measurements of the environmental temperature, humidity and the sows’ activity level and respiratory rate were conducted approximately every two hours by visual monitoring. The environmental temperature and humidity were measured at two different locations in the farrowing units. The measuring devices (Indoor Hygrometer/thermometer, Model E0119TH, Co-tech Development Corporation, Taiwan, R.O.C.) were placed close to the second or third pen into the room to avoid the exterior walls, approximately 60–70 cm above the floor. Activity level was recorded as 1: lying down (lateral or sternal recumbency), 2: sitting position, 3: standing position or 4: rooting or nest building behaviour (restlessness and manipulation of nest-building material such as straw or hay). Respiratory rate was recorded by observation and counting of flank movements for one minute.

### Repeated temperature measurements

**Fig. 4:**
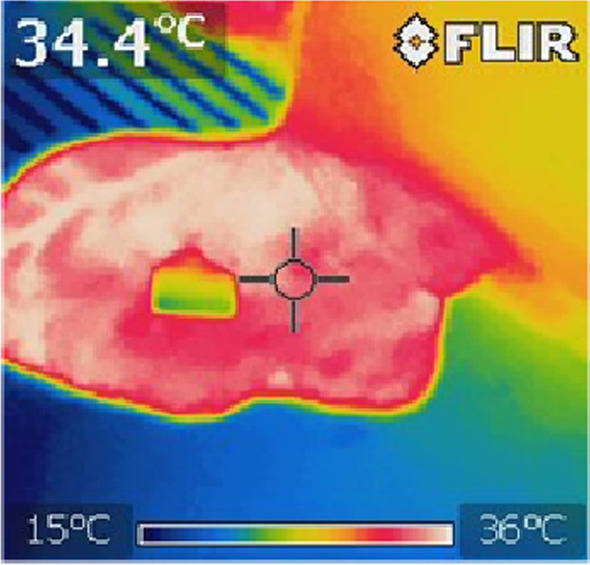
Location of the auricle centre point for the use of infrared camera. Legend: Location of the auricle centre point (within the sighting target) which was used for temperature determination by infrared camera in sows in three Norwegian pig farms. A deviation of 1–2 cm from the centre point was accepted

The sow’s rectal temperature was measured by a digital rectal thermometer (RT), (DIGI-TEMP, KRUUSE, 1440 Drøbak, Norway) inserted approximately eight centimeters into the rectum of the sow three times per day. A mark was placed on the thermometer to ensure the same depth of rectal intrusion in all animals. The thermometer was inserted into the anus and positioned in contact with rectal mucosa, until hearing an acoustic signal, in accordance with the manufacturer’s instructions. Temperature measurement of the outer skin surface of the auricle was performed by using the infrared camera (IRC) FLIR i7 (FLIR Systems, Inc 27,700 SW Parkway Ave. Wilsonville, OR 97,070 USA) from a distance of 30 to 50 cm according to the manufacturer’s recommendations. Temperature measurements by IRC were evaluated by computer analysis (FLIR Tools), enabling location of the auricle centre point which was used for temperature determination (Fig. 4). From the same auricle centre point, skin surface temperature was measured by infrared laser thermometer (IRT) (Sentry St 653 laser thermometer, Sentry Optronics Corp, Sammin Road, Ban-Ciao, Taipei 220, Taiwan, R.O.C.) parallel to the IRC measurements. Auricular skin- and rectal temperature measurements were conducted approximately every two hours. At Farms 1 and 2, the auricular skin temperature of one ear was registered. If the sow was lying down, the one ear facing upwards was measured. If the sow was standing, the right or the left ear was measured. At Farm 3, both ears were measured each time. For the statistical analysis, the highest temperatures of the left or right ear were used. Single measurements of body temperature > 42 °C (n = 27) and below 20 °C (n = 1) were excluded from further statistical analyses, as they were considered either to be from sows with a potential fever or due to measurement errors, respectively.

### Statistics

Data recorded were stored in Microsoft Excel (Microsoft Corporation, Redmond, WA) and transferred to Stata (STATA SE/15 for Windows; Stata Corp., College Station, TX, USA) for descriptive and statistical analyses. The final dataset from 91 sows in three farms included 2442 IRC temperatures, 2456 IRL temperatures and 1471 RT temperatures measured from 48 h before farrowing to 24 h post farrowing.

First, a descriptive analysis of the observed data, such as respiration rate and activity, and the temperatures measured using the three different methods, were graphically explored. Two-way plots, with the polynomial function in STATA was used for the graphical exploration to visualize the different temperatures measured over time. This “lpoly” function was used to perform a kernel-weighted local polynomial univariate regression of respiration rate, mean activity, and temperature on the y-axis and time on the x-axis.

In addition, a Pearson correlation was used to investigate the relationship between the observed temperature data over time and if skin and rectal temperatures were associated with room temperature and respiration rates.

Secondly, statistical models were developed to estimate coefficients of the auricular skin temperature and rectal temperature of the sows in the period before, at, and after farrowing. The time before and after farrowing was divided into ten timeslots, four prior to and five post farrowing.

Three two-level mixed models were then fitted for the outcomes (1) RT, (2) IRC and (3) IRT. In all models, sow was included as random effect, accounting for the dependency within each sow. Dependence between measured temperatures was accounted for by including the time categories (ten timeslots). Farm was also included as a fixed effect, accounting for farm specifics in management, climate conditions (temperature and humidity) and pen design.

In all three models, normality plots of standardized residuals were evaluated, and potential outliers and observations with large influence were explored. The model was tested with and without possible influencing points and the best-fitted models were used.

To investigate the possibility of using infrared thermography to estimate the onset of parturition in sows, the results from these models were presented in figures showing the predictive margins of temperature on time with a pointwise 95% confidence interval (CI) (bars) using the command marginplots in STATA. Temperatures with no overlap in CI were interpreted as having a significant difference in mean value per time point.

## Data Availability

The datasets used during the current study are available in an anonymized form from the corresponding author on reasonable request.
